# Secretome profiling of human epithelial cells exposed to cigarette smoke extract and their effect on human lung microvascular endothelial cells

**DOI:** 10.1038/s41598-024-64717-x

**Published:** 2024-06-14

**Authors:** Porrnthanate Seenak, Nitirut Nernpermpisooth, Sarawut Kumphune, Worawat Songjang, Arunya Jiraviriyakul, Noppadon Jumroon, Panyupa Pankhong, Sittiruk Roytrakul, Siriwan Thaisakun, Narumon Phaonakrop, Nitra Nuengchamnong

**Affiliations:** 1 Integrative Biomedical Research Unit (IBRU), Faculty of Allied Health Sciences, Naresuan University, Phitsanulok, 65000 Thailand; 2 Department of Cardio-Thoracic Technology, Faculty of Allied Health Sciences, Naresuan University, Phitsanulok, 65000 Thailand; 3 Biomedical Engineering and Innovation Research Centre, Chiang Mai University, Mueang, Chiang Mai, 50200 Thailand; 4 Biomedical Engineering Institute, Chiang Mai University, Mueang, Chiang Mai, 50200 Thailand; 5 Department of Medical Technology, Faculty of Allied Health Sciences, Naresuan University, Phitsanulok, 65000 Thailand; 6 National Centre for Genetic Engineering and Biotechnology (BIOTEC), National Science and Technology Development Agency, Pathum Thani, 12120 Thailand; 7 Science Laboratory Center, Faculty of Science, Naresuan University, Phitsanulok, 65000 Thailand

**Keywords:** Cigarette smoke, Pulmonary disease, Lung epithelial cell secretome, Lung microvascular endothelial cell, Secretome profiling, Inflammation, Cell biology, Medical research, Molecular medicine

## Abstract

Cigarette smoke (CS) is one of the leading causes of pulmonary diseases and can induce lung secretome alteration. CS exposure-induced damages to human pulmonary epithelial cells and microvascular endothelial cells have been extensively demonstrated; however, the effects of the secretome of lung epithelial cells exposed to CS extracts (CSE) on lung microvascular endothelial cells are not fully understood. In this study, we aimed to determine the effects of the secretome of lung epithelial cells exposed to CSE on lung microvascular endothelial cells. Human lung epithelial cells, A549, were exposed to CSE, and the secretome was collected. Human lung microvascular endothelial cells, HULEC-5a, were used to evaluate the effect of the secretome of A549 exposed to CSE. Secretome profile, endothelial cell death, inflammation, and permeability markers were determined. CSE altered the secretome expression of A549 cells, and secretome derived from CSE-exposed A549 cells caused respiratory endothelial cell death, inflammation, and moderately enhanced endothelial permeability. This study demonstrates the potential role of cellular interaction between endothelial and epithelial cells during exposure to CSE and provides novel therapeutic targets or beneficial biomarkers using secretome analysis for CSE-related respiratory diseases.

## Introduction

Cigarette smoke (CS) is a strong risk factor associated with the development of various systemic diseases, including metabolic and cardiovascular diseases, stroke, and conditions related to pregnancy and birth^1^. It also contributes to respiratory diseases, such as asthma, chronic obstructive pulmonary disease, chronic bronchitis, and emphysema, as well as cancer^2^. CS contains several toxicants that can induce lung epithelial and endothelial cell death^3,4^ by elevating oxidative stress and inflammation^5–7^.

The respiratory membrane mainly comprises alveolar epithelial cells and interstitial matrix surrounded by microvascular endothelial cells^8^. The alveolar–capillary barrier regulates gas exchange, infection, and permeability homeostasis^9^. CS can initiate alveolar–capillary barrier dysfunction, leading to vascular leakage^10^. Smokers often exhibit impaired permeability of the pulmonary capillary barrier and reduced endothelial cellular junctions^10,11^. In addition, CS can directly damage tight junction proteins, increase lung epithelial permeability^12^, and promote the secretion of endothelial cytokines, chemokines, and secretomes^13^. This highlights that the epithelium and endothelium may have crosstalk pathways and molecular connections during exposure to CS. However, the molecular mechanism underlying the effect of secretome derived from lung epithelial cells on lung endothelial cells has not been investigated in detail. This study aimed to determine the effects of CS extract (CSE) on lung epithelial cell secretome and its effects on lung microvascular endothelial cells and identify the secretome profile of human lung epithelial cells exposed to CSE.

## Results

### Effects of CSE on the viability of human lung epithelial cells

**Figure 1:**
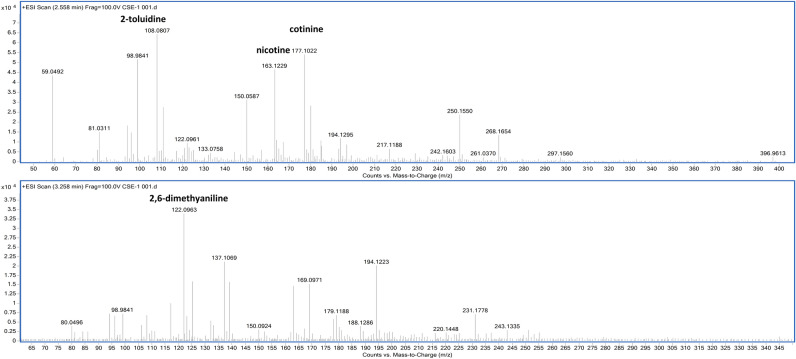
Analysis of the chemical composition of CSE using MS/MS.

**Figure 2:**
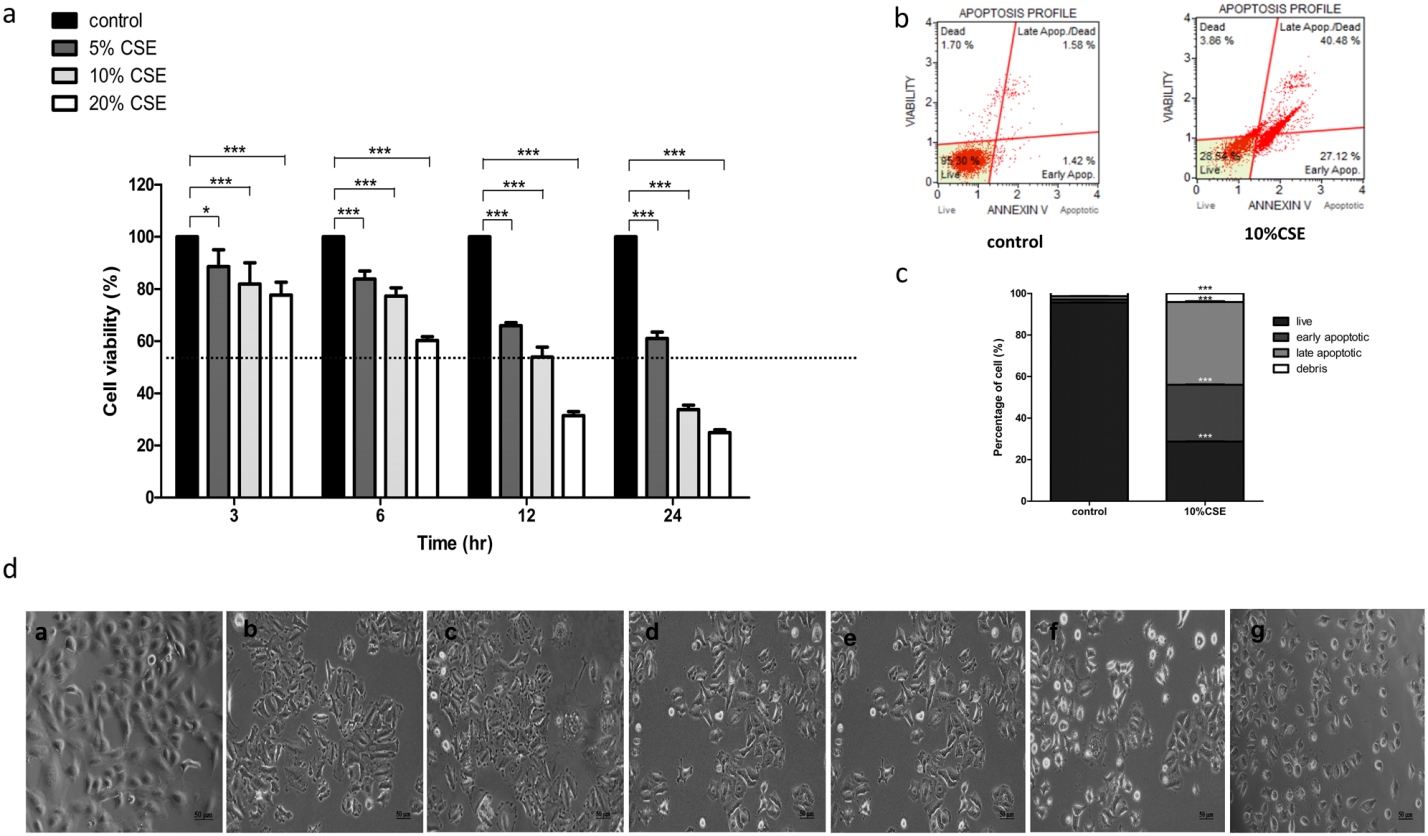
Effects of CSE on A549 cell viability, cell death and cell morphology. The finding demonstrated CSE includes lung epithelial cell death in a dose- and time-dependent manner, and 10% CSE caused cell morphology alteration. (**a**) A549 cells were treated with CSE at different doses for various time periods. Cell viability was determined using an MTT assay. (**b**) A549 cell death evaluated using Annexin V/PI staining. (**c**) Percentage of cell death after exposure to 10% CSE. d. Control (a) and A549 cells were treated with 10% CSE for 3 h (**b**), 6 h (**c**), 12 h (**d**), 24 h (**e**), 36 h (**f**), and 48 h (**g**).

Tandem mass spectroscopic analysis was performed to validate the method of CSE extraction. Our findings revealed that CSE was composed of nicotine, cotinine, 2-toluidine, and 2,6-dimethylaniline (Fig. 1). CSE (10%) significantly reduced the viability of A549 cells to 53.86 ± 3.86% (IC_50_) after 12 h of treatment (*p* < 0.05, Fig. 2a). Cells treated with 10% CSE had significantly higher percentages of early apoptosis (27.327 ± 0.168 vs. 1.440 ± 0.031*, p* < 0.05), late apoptosis (39.840 ± 0.364 vs. 1.587 ± 0.052*, p* < 0.05), and debris (4.127 ± 0.184 vs. 1.367 ± 0.173*, p* < 0.05) than the untreated control cells (Fig. 2b,c). Moreover, 10% CSE caused morphological alterations of human lung epithelial cells (Fig. 2d). These data suggest that CSE induces the death of A549 cells and alters their morphology in a dose- and time-dependent manner.

### The secretome of A549 cells exposed to CSE promotes inflammation and death of human lung microvascular endothelial cells

**Figure 3:**
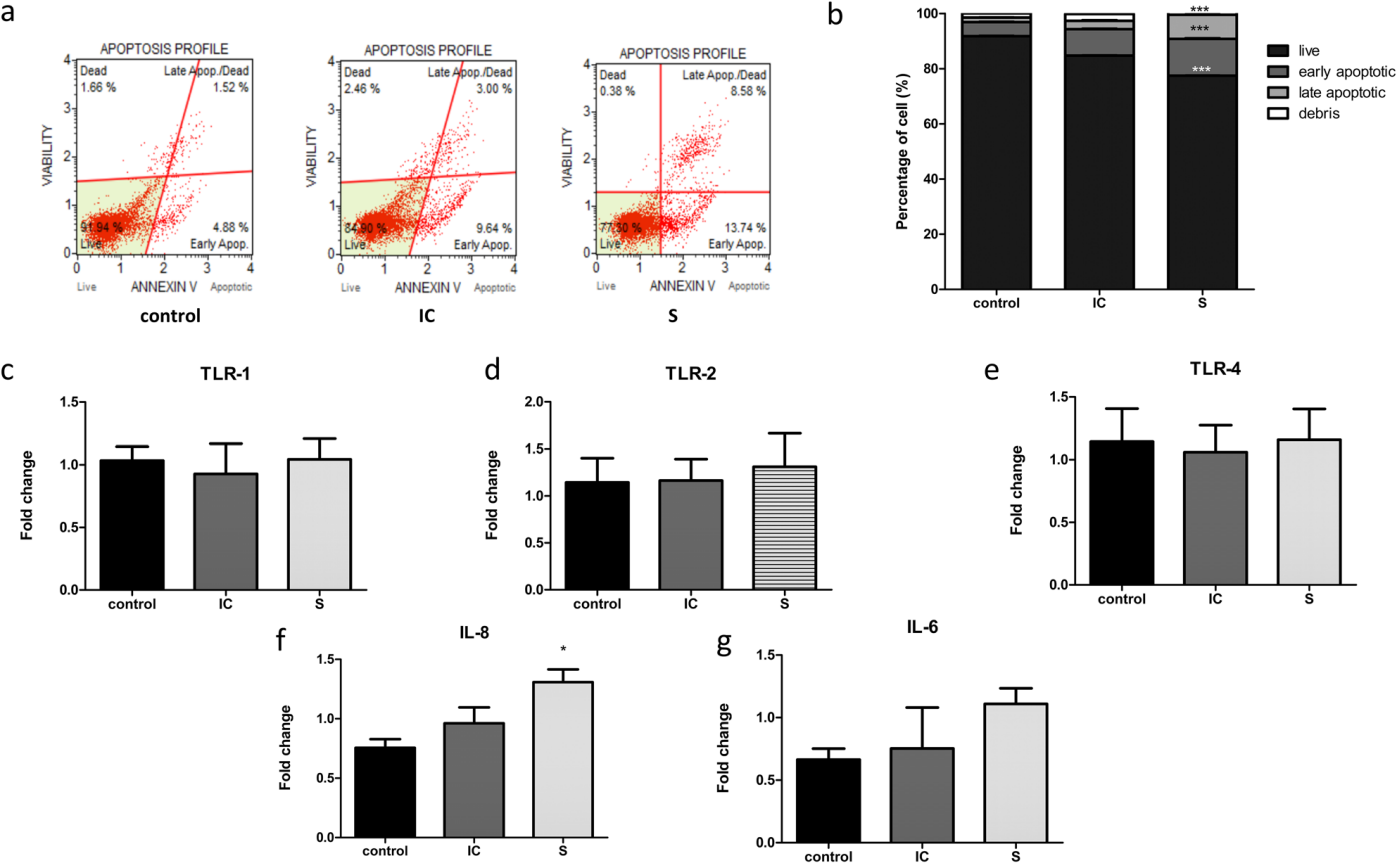
Effects of the secretome derived from A549 cells exposed to CSE on HULEC-5a cell death and inflammation. Lung endothelial cells (HULEC-5a) subjected to CS-secretome derived from A549 cells significantly reduced cell viability and increased IL-8 expression. (**a**) HULEC-5a cells were treated with the secretome for 6 h. Cell death was determined using Annexin V/PI staining. (**b**) Percentage of cell death after exposure to the secretome. Expression of (**c**) TLR-1, (**d**) TLR-2, (**e**) TLR-4, (**f**) interleukin-6 (IL-6), and (**g**) interleukin-8 (IL-8). Data are presented as mean ± SEM. *p < 0.05 vs. the control group.

Annexin V/propidium iodide (PI) staining showed that the secretome group had a significantly higher percentage of early apoptosis (13.38 ± 0.2 vs. 5.12 ± 0.029,* p* < 0.05), late apoptosis (8.733 ± 0.116 vs. 1.633 ± 0.094,* p* < 0.05), and decreased cell viability (77.433 ± 0.068 vs. 91.767 ± 0.135,* p* < 0.05) than the control group (Fig. 3a,b). We performed quantitative reverse transcription-polymerase chain reaction (RT-PCR) analysis to determine the expression of the proinflammatory cytokine gene *IL6*, proinflammatory chemokine gene *IL8*, and Toll-like receptor genes *TLR1*, *TLR2*, and *TLR4*. The S group (cells cultured in the collected secretome) had a significantly higher level of *IL8* than the control group (*p* < 0.05) and an increasing trend of *IL-6* expression (Fig. 3f,g), while no significant changes in the expression of *TLR1*, *TLR2*, and *TLR4* were noticed (Fig. 3c–e). These results indicate that the secretome of A549 cells exposed to CSE had a direct effect on inflammation and death of human respiratory microvascular endothelial cells.

### The secretome of A549 exposed to CSE alters the integrity of human lung microvascular endothelial cells and the expression of caveolin-1 and claudin-5

**Figure 4:**
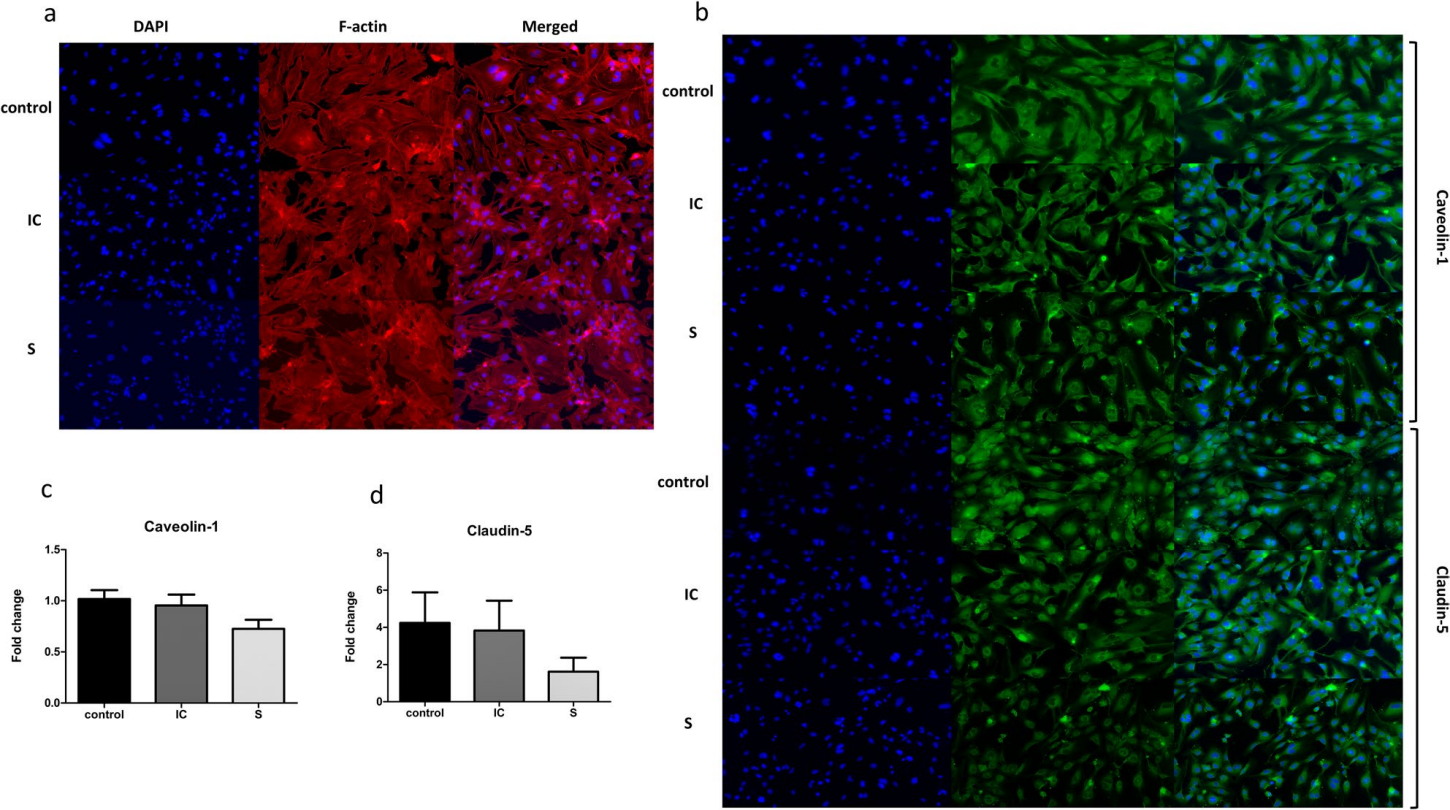
Effects of the secretome of A549 cells exposed to CSE on HULEC-5a cell integrity and viability. Secretomes derived from lung epithelial cells exposed to CSE reduce the integrity of the cells and increase the permeability of the respiratory endothelial cell. (**a**) Staining of actin cytoskeleton. (**b**) Immunofluorescence staining of caveolin-1 and claudin-5. (**c**) Caveolin-1 expression. (**d**) Claudin-5 expression. Data are presented as mean ± SEM. *p < 0.05 vs. the control group.

Inflammation and death of respiratory endothelial cells can induce the dysfunction of the vascular permeability barrier. The integrity and permeability of human lung microvascular endothelial cells were evaluated by staining HULEC-5a cells with phalloidin-iFlour 555, caveolin-1, and claudin-5. HULEC-5a treated with the secretome of A549 cells exposed to CSE showed actin disorganization, loss of stress fiber, and altered morphology (Fig. 4a). In addition, we observed a moderate reduction of caveolin-1 and claudin-5 expression in the S group (Fig. 4b–d). These observations suggest that the secretome of respiratory epithelial cells can contribute to a weakened lung membrane integrity and may enhance lung microvascular permeability.

### Analysis of the secretome of A549 cells exposed to CSE

**Figure 5:**
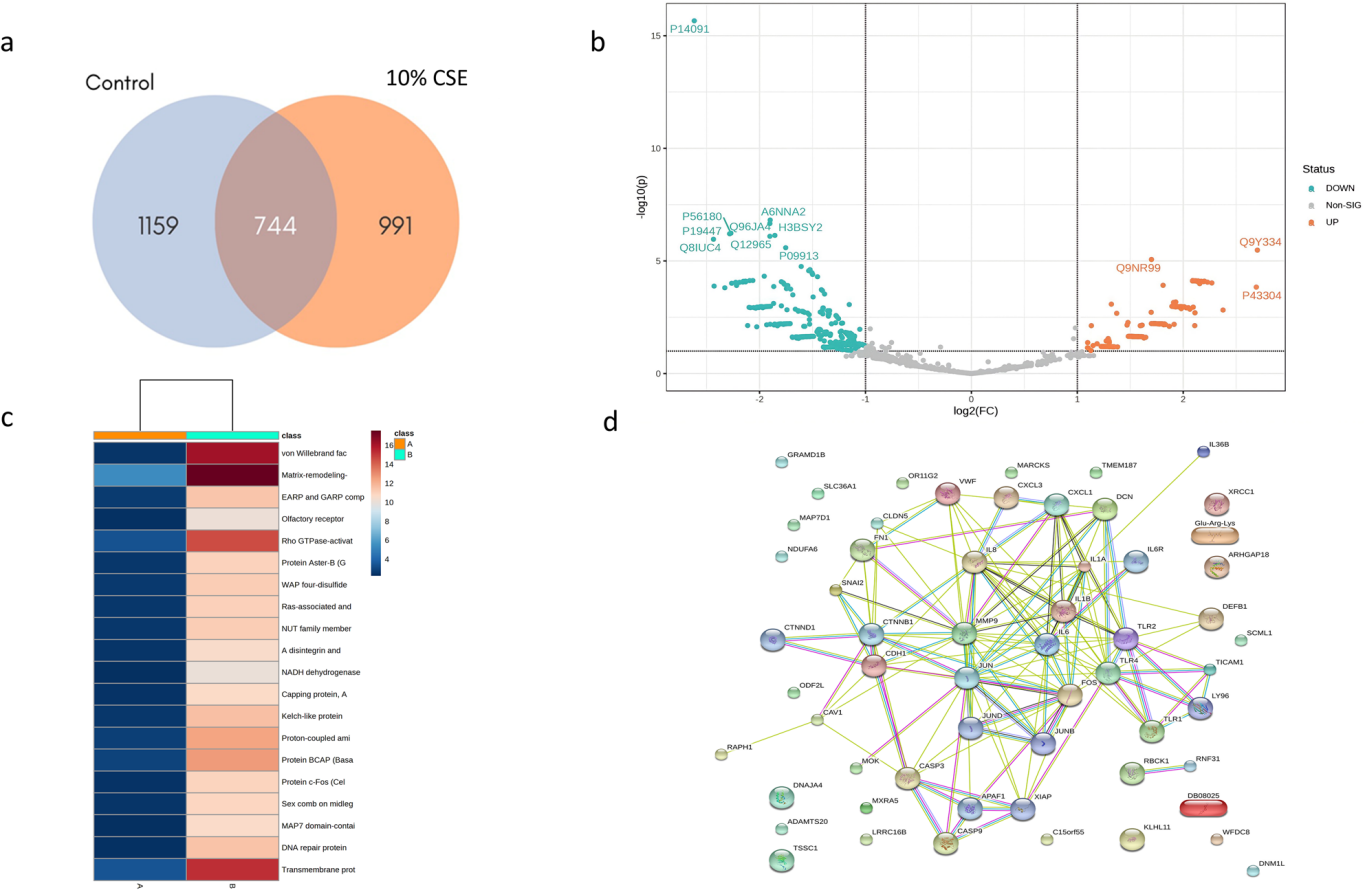
The secretome of A549 cells exposed to CSE and possible pathways of respiratory epithelial–endothelial interaction during CSE exposure—correlation of inflammation mechanism, cell death pathway, and actin reorganization process. (**a**) Venn diagram; (**b**) volcano plot; (**c**) heatmap of top 20 proteins; (**d**) protein–protein interaction by STRING analysis.

The secretome data of human epithelial cells exposed to CSE identified a total of 2,894 proteins, among which 991 were identified to be unique in the secretome of the 10% CSE group; and 1,159 proteins were unique in the secretome of untreated A549 cells (Supplementary Materials; Table S1, Fig. 5a). Venn diagrams showed 278 upregulated proteins (*p* < 0.1) with respect to those of the control group (Fig. 5b). Ontological analysis of the identified proteins revealed their association with several functions, such as biological process involved in interspecies interaction between organisms (23.68%), immune system process (12.95%), protein localization (6.52%), and metabolic process (2.82%). Twenty top upregulated proteins were determined as shown in Table 1 and Fig. 5c. We identified von Willebrand factor A domain-containing protein 7 (VWA7) and Rho GTPase-activating protein 18 as crucial players in vascular biology, three proteins related to inflammation (Kelch-like protein 11, c-FOS, and transmembrane protein 187), two proteins involved in extracellular matrix remodeling (matrix remodeling-associated protein 5 and A disintegrin and metalloproteinase with thrombospondin motifs 20), and three proteins associated with actin and cytoskeleton [Ras-associated and pleckstrin homology domains-containing protein 1 (RAPH1), Capping protein, Arp2/3 and myosin-I linker protein 3, and MAP7 domain-containing protein 1). Furthermore, we identified three damage-associated molecular patterns (DAMPs), including decorin, heat shock protein-40, and beta-defensin, among 278 upregulated proteins. Further investigation showed that five dominant proteins (decorin, VWA7, c-FOS, beta-defensin, and RAPH1) are possibly involved in the extracellular matrix remodeling pathway, inflammation, cell death, and endothelium permeability pathway (Fig. 5d).

**Table 1:** Top twenty upregulated proteins in the secretome of A549 cells exposed to CSE.

Protein name	Accession number	Gene Ontology	Log _2_(FC)
von Willebrand factor A domain-containing protein 7	Q9Y334	Protein folding	2.7004
Matrix-remodeling-associated protein 5	Q9NR99	Cellular component 1. Extracellular region	1.7005
EARP and GARP complex-interacting protein 1	Q53HC9	Biological process 1. Protein ubiquitination	2.0897
Olfactory receptor 11G2	Q8NGC1	Protein folding	2.1124
Rho GTPase-activating protein 18	Q8N392	Biological process 1. Regulation of actin filament polymerization 2. Actin filament polymerization Molecular function 1. Protein binding 2. GTPase activity 3. GTPase activator activity	2.1206
Protein Aster-B	Q3KR37	Biological process 1. Intracellular sterol transport Molecular function 1. Sterol binding 2. Lipid transporter activity	2.1214
WAP four-disulfide core domain protein 8	Q8IUA0	Protein folding	2.0976
Ras-associated and pleckstrin homology domains-containing protein 1	Q70E73	Cellular component 1. Cytosol 2. Plasma membrane	2.115
NUT family member 1	Q86Y26	Protein folding	2.141
A disintegrin and metalloproteinase with thrombospondin motifs 20	P59510	Biological process 1. Extracellular matrix organization Molecular function 1. Metalloendopeptidase activity	2.0882
NADH dehydrogenase [ubiquinone] 1 alpha subcomplex subunit 6	P56556	Response to oxidative stress	2.1136
Capping protein, Arp2/3 and myosin-I linker protein 3	Q8ND23	Biological process 1. Actin nucleation actin cytoskeleton reorganization 2. Cell migration	2.1262
Kelch-like protein 1	Q9NVR0	Protein folding	2.1998
Proton-coupled amino acid transporter 1	Q7Z2H8	Biological process 1. Proton transmembrane transport 2. Amino acid transmembrane transport Molecular function 1. Neutral amino acid transmembrane transporter activity 2. Organic anion transmembrane transporter activity 3. L-amino acid transmembrane transporter activity	2.169
Protein BCAP	Q9ULJ1	Biological process 1. Intraciliary transport involved in cilium assembly 2. Negative regulation of organelle organization 3. Regulation of cell projection assembly 4. Regulation of plasma membrane-bounded cell projection organization	2.1347
Protein c-Fos	P01100	Biological process 1. Transcription by RNA polymerase II 2. Regulation of transcription by RNA polymerase II Molecular function 1. DNA-binding transcription factor activity, RNA polymerase II-specific 2. RNA polymerase II cis-regulatory region sequence-specific DNA binding	2.1922
Sex comb on midleg-like protein 1	Q9UN30	Protein folding	2.1372
MAP7 domain-containing protein 1	Q3KQU3	Microtubule cytoskeleton organization	2.2294
DNA repair protein XRCC1	P18887	Protein folding	2.2701
Transmembrane protein 187 (Protein ITBA1)	Q14656	Cellular component 1. Transport vesicle 2. Plasma membrane 3. Vacuole	2.1558

*FC* Fold change.

## Discussion

CS is a major cause of human respiratory epithelial cell death, resulting in the secretion of several proteins, which finally leads to lung microvascular endothelial injury. The present study highlights that the secretome of human lung epithelial cells exposed to CSE can promote inflammation of human respiratory endothelial cells and moderately enhance vascular permeability. In addition, we showed the possible protein–protein interactions in this study.

CS contains several toxicants, such as nicotine, cotinine, 2-toluidine, and 2,6 dimethylaniline^3,4^. Previous preclinical and clinical studies have shown that CS can promote cell death, particularly in lung epithelial cells^14–18^. Consequently, several mechanisms underlying the effects of CS have been identified, including the direct promotion of oxidative stress, injury, and inflammation. Direct or indirect exposure to CS can disrupt the balance between oxidative stress and antioxidative systems by modulating glutathione, superoxide dismutase, and glutathione peroxidase activities^15^. Moreover, CS can directly induce superoxide anion generation, leading to oxidative stress-induced tissue damage^16^. Overproduction of reactive oxygen species triggers an intracellular signaling cascade that promotes pro-inflammatory mediator expression^17^. Oxidative stress and inflammation dysregulation are key active players that contribute to the activation of cell death^18^. Several studies have reported that CS significantly induces necrosis and alters the secretome of lung epithelial cells^19–21^. In the present study, human lung epithelial cells exposed to CS exhibited cytotoxicity and necrotic cell death in a dose- and time-dependent manner, along with alteration in secretomic profile. Our results revealed varying percentages of cell death between the two methods (MTT and Annexin V/PI assay), reflecting the different levels of cellular mechanisms detected via both methods.

**Figure 6:**
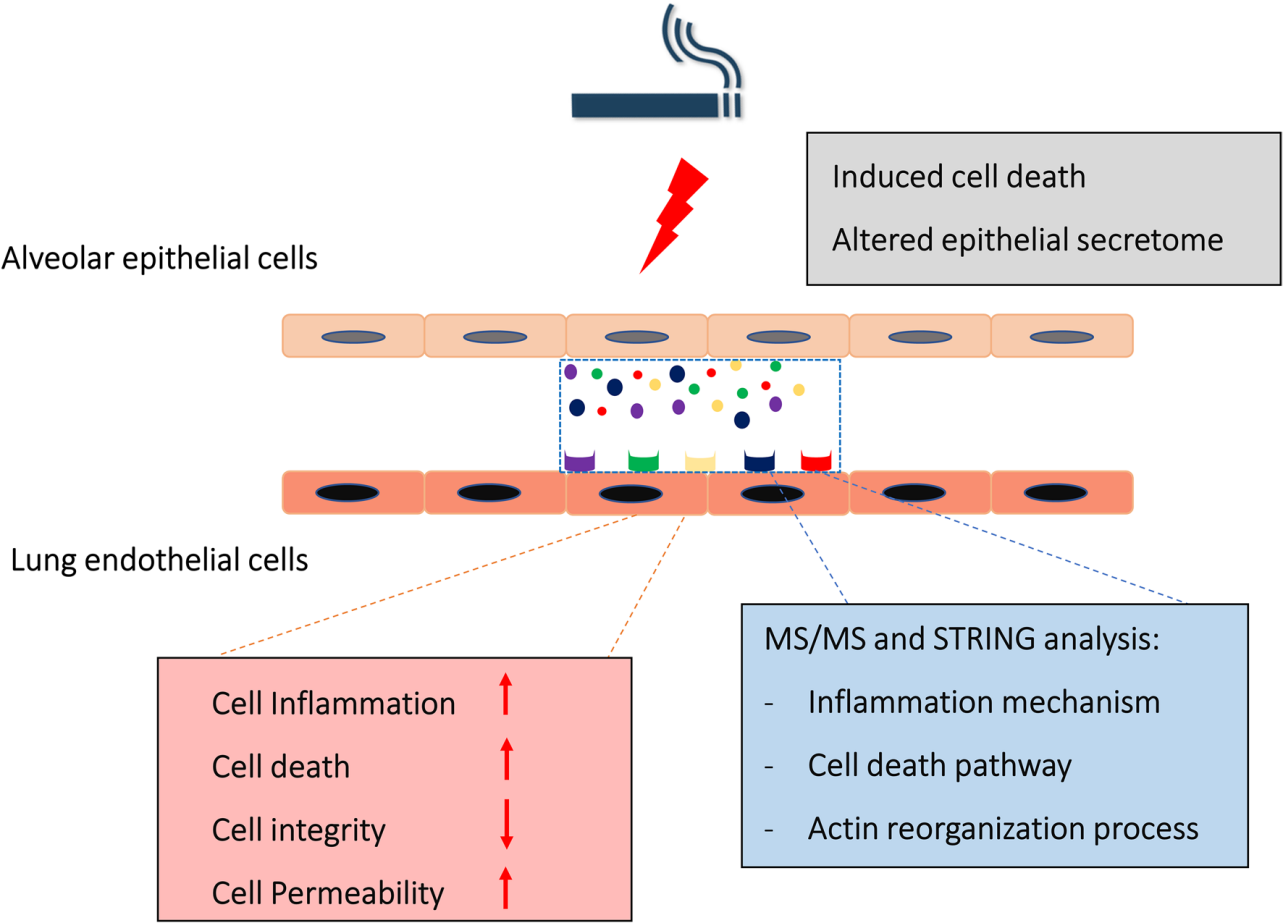
The postulate mechanism of respiratory epithelial–endothelial interaction during CSE exposure. CSE induces lung epithelial secretome alterations. Candidate proteins (VWA7, c-FOS, beta-defensin, decorin, and RAPH1) related to respiratory endothelial inflammation, death and reorganization of actin by STRING analysis.

Normally, alveolar epithelial and vascular endothelial cells form a semipermeable interface, which preserves the homeostasis of pulmonary microcirculation and maintains alveolar–capillary interaction^8,22^. Alteration of secretory proteins in the respiratory layer impairs its function. Several preclinical and clinical studies have reported that CS can alter respiratory secretome, leading to variation in epithelial–endothelial cell communication^10–12,22^. This secretory secretome can promote endothelial cell injury^10,21^. Although respiratory endothelial cell injury by direct CS exposure has been reported^2,7,21^, the molecular mechanism underlying the interaction between lung epithelial and endothelial cells during CS exposure has not been fully investigated, and the key role of endothelial cells during CS exposure is largely unexplored. The present study demonstrated, for the first time, the molecular mechanism of CS-exposed secretome derived from respiratory epithelial cells in pulmonary microvascular endothelial cells (Fig. 6).

Our findings showed that the CSE-exposed secretome significantly induced *IL8* expression and reduced cell viability in the S group. These findings are consistent with those of a previous study^23^ and suggest that secretome derived from CS-exposed lung epithelial cells can induce lung endothelial cell inflammation and cell death. IL-8, a proinflammatory chemokine and an autocrine/paracrine growth factor is a key player in neutrophil recruitment to the inflammation site and plays a crucial role in endothelial cell survival, proliferation, and angiogenesis^24,25^. Damaged respiratory endothelial cells secrete inflammatory mediators, which can further enhance inflammation, leading to endothelial cell death. Concordant with this, Demedts et al. have demonstrated lung endothelial cell death caused by CS-induced inflammation in patients^26^. These findings indicate a key role for lung endothelial cells in response to CS exposure.

The lung microvascular endothelial cell barrier forms tight and close contacts that regulate the movement of various molecules and leukocytes into the interstitium and subsequently into the respiratory air spaces^27^. A reduction in the integrity of lung microvascular endothelial cells results in increased endothelial permeability, enhancing inflammation, inducing pulmonary edema, and progressing lung diseases^28^. Our results showed that lung endothelial cells treated with CS-exposed secretome derived from respiratory epithelial cells exhibited actin reorganization, moderately reduced caveolin-1 and claudin-5 expression and slightly decreased *caveolin-1* and *claudin-5* mRNA expression. These observations suggest that incubation for 6 h may be late for the detection of *caveolin-1* and *claudin-5* mRNA expression. However, under physiological conditions, the epithelial–endothelial membrane interaction is uninterrupted. Caveolin-1, a transmembrane protein, and claudin-5, a tight junction protein, both of which are abundant in lung endothelial cells, play important roles in regulating vascular permeability^29,30^. *Cav1*^*−/−*^ mutation can increase extravascular lung water by limiting water transport, thereby leading to pulmonary edema^31^, while overexpression of claudin-5 reverses oxygenation and modulates vascular leakage in a rat model of acute respiratory distress syndrome^32^. Therefore, the CS-exposed secretome of lung epithelial cells may increase vascular permeability. Several studies reported an increase in IL-8 expression in CSE exposure, which promotes protease enzyme activity and expression^23,33^. This increase in protease enzyme activity can lead to endothelial cell membrane destruction and loss of the actin cytoskeleton. However, the specific role of protease enzyme activity in endothelial permeability requires further investigation.

Analysis of epithelial secretome revealed the five candidate proteins, including VWA7, c-FOS, beta-defensin, decorin, and RAPH1. Prediction of protein–protein interaction of secretome and endothelial receptors, particularly in Toll-like receptors (TLRs) 1, 2 and 4, demonstrated that these candidate proteins play important roles in several mechanisms of endothelial cells, especially those related to inflammation, cell death pathway, and actin reorganization. In this study, secretome profiles and candidate proteins of lung epithelial cells exposed to CSE were explored, revealing that the secretome can induce lung microvascular endothelial cell inflammation and increase vascular lung permeability. These results suggest a strong association between the predicted pathways and our previously mentioned results. These findings suggest novel targets for the treatment of CS-related respiratory diseases as well as potential biomarkers. However, the current study was performed only under 2D indirect co-culture conditions; therefore, further studies using an air–liquid interface system are required to support these findings.

Conclusively, the present study demonstrates the in vitro effects of secretome released from human alveolar epithelial cells exposed to CSE on human microvascular endothelial cells and reveals novel therapeutic targets and potential biomarkers. Nevertheless, the precise functions and underlying mechanisms of the identified candidate proteins and pathways in smoking-related diseases should be further validated in both preclinical and clinical studies.

## Methods

### Cell culture

The human lung epithelial cell line A549 (CCL-185) and human lung microvascular endothelial cell line HULEC-5a (CRL-3244) were purchased from the American Type Culture Collection (). A549 cells were cultured in RPMI-1640 (Thermo Fisher Scientific, USA) supplemented with 10% fetal bovine serum (FBS; Thermo Fisher Scientific). HULEC-5a cells were cultured in MCDB131(Thermo Fisher Scientific) supplemented with growth medium, containing 10 ng/mL epidermal growth factor (PHG0314; Thermo Fisher Scientific), 1 µg/mL hydrocortisone (PHG0314; Sigma-Aldrich, St. Louis, MO, USA), 10 mM glutamine (ATCC30-2214), and 10% FBS. Cells were maintained at 37 °C in a water-saturated atmosphere of 5% CO_2_ and 95% air.

### Preparation and composition analysis of CSE

CSE was prepared using commercial cigarettes (Marlboro, Philip. Morris, Inc., Richmond, VA, USA), and mainstream smoke was bubbled through 1 mL/cigarette of RPMI-1640 medium using 50-mL glass syringes with glass plungers (50 mL puffs every 10 s), followed by pH adjustment (7.35–7.45). CSE was filtered through a 0.22-µM filter and used for further investigation. The CSE solution was considered to be 100%. To determine the chemical components of CSE, LC-ESI-QTOF 6540 (Agilent Technologies, Singapore) coupled with an Agilent 1260 infinity Series HPLC system (Agilent, Waldbronn, Germany) was used. The separation was performed with the Luna C18 column (4.6 × 150 mm, 5 µm; Phenomenex, USA) at a flow rate of 500 µL/min and control temperature of 35 °C. Water type I (Millipore, USA) with 0.1% (v/v) formic acid and acetonitrile B with 0.1% (v/v) formic acid were used as mobile phases A and B, respectively. The gradient elution mode started with 5% solvent B to 95% B linear gradient within 30 min and held on at this ratio for 10 min, followed by a 5-min post-run for column equilibrium. The injection volume was 5 µL. The operating parameters in MS detection were as follows: drying gas, N_2_; flow rate, 10.0 L/min; drying gas temperature, 350 °C; nebulizer pressure, 30 psig; capillary, 3500 V; skimmer, 65 V; octapole RFV, 750 V; and fragmentor voltage, 100 V. The fragmentation MS/MS mode was set up at 3 collision energies of 10, 20, and 40 V using high-purity nitrogen gas (99.999%) as the collision gas. The mass range was set at m/z 50–400 with 250 ms/spectra controlled by Agilent LC–MS-QTOF Mass Hunter Data Acquisition Software version B.05.01 and Agilent Mass Hunter Qualitative Analysis Software B 06.0 (Agilent Technologies, USA). All the acquisition and analysis of the data were performed in the positive ionization mode. The compounds identified based on accurate mass and fragmentation patterns were compared with those in public databases, such as the Human Metabolome Database (HMDB) and the METLIN Metabolomics Database and Library (Agilent Technology, USA).

### Determination of cell viability and death

A549 cells were seeded in 96-well plates and cultured in the absence and presence of 5%, 10%, and 20% CSE at different time points (3, 6, 12, and 24 h). Cells were incubated with MTT solution (0.01 g/mL) for 2 h to measure cell viability. The formazan crystals were dissolved in dimethyl sulfoxide, and absorbance at 490 nm was measured using an EnSpire multimode plate reader (PerkinElmer). The optical density of the control group was considered 100% cell viability. Cell death was analyzed via Annexin V/PI staining using a Muse analyzer (Sigma-Aldrich), according to the manufacturer’s protocols.

### Secretome collection and culture protocol

A549 cells were cultured in a cell culture dish with or without 10% CSE for 12 h. Cells were washed twice with phosphate-buffered saline (PBS), and RPMI-1640 (serum-free media) was replaced, followed by incubation for 12 h. The secretome was collected and kept at − 80 °C until analysis. To determine the effects of secretome derived from A549 cells exposed to CSE, HULEC-5a cells were divided into three groups, including control (cultured in complete MCDB131; C), internal control (cultured in serum-free RPMI-1640 medium; IC), and secretome (cultured in collected secretome; S). Cells of each group were cultured for 6 h.

### Secretome analysis using liquid chromatography-tandem mass spectrometry (LC–MS/MS)

After incubation, the secretome was collected and transferred to a new tube, precipitated, and centrifuged, and the pellet was collected. It was dried and kept at − 80 ℃ prior to use. Protein concentrations of secretome samples were determined using the Lowry assay method using bovine serum albumin (BSA) as an internal control^34^. Five micrograms of the samples was reduced, alkylated, and digested using sequencing-grade porcine trypsin (1:20 ratio) at 37 ºC for 16 h. After digestion, the tryptic peptides were vacuum-concentrated and resuspended in 0.1% formic acid for nano LC–MS/MS analysis.

To perform LC/MS–MS, 100 µL of tryptic peptides was enriched on a µ-Precolumn (300 µm i.d. × 5 mm C18 Pepmap 100, 5 µm, 100 A; Thermo Scientific, UK), separated on a 75 μm i. d. × 15 cm, and packed within Acclaim PepMap RSLC C18, 2 μm, 100 Å, nanoViper (Thermo Scientific). The mobile phase comprised solvents A (0.1% formic acid in water) and B (0.1% formic acid in 80% acetonitrile), and peptides were fractionated using 5–55% solvent B at a constant flow rate of 0.30 μL/min for 30 min and then loaded onto a Ultimate3000 Nano/Capillary LC System (Thermo Scientific) coupled to a ZenoTOF 7600 mass spectrometer (SCIEX, Framingham, MA, USA).

The source and gas parameters of the ZenoTOF 7600 system for all acquisitions were set as follows: ion source gas 1, 8 psi; curtain gas, 35 psi; CAD gas, 7 psi; source temperature, 200 °C; polarity, positive; and spray voltage, 3300 V.

### Bioinformatic analysis of the secretome

The MS data were analyzed using MaxQuant v.2.2.0.0 with the Andromeda search engine to quantify proteins that correlated with those in the UniProt *Homo sapiens* database^35^. The criteria for label-free quantitation using the MaxQuant standard were set as follows: maximum of two miss cleavages, mass tolerance (0.6 Da), enzyme (trypsin), fixed modification (carbamidomethylation of cysteine), and variable modifications (oxidation of methionine and acetylation of the protein N-terminus). Only peptides with a minimum of seven amino acids and at least one unique peptide were required for protein identification. The false discovery rate was set at 1%. The MaxQuant ProteinGroups.txt file was submitted into Perseus v.1.6.6.0^36^. Maximum intensities were transformed into log_2_, and *t*-tests were conducted to compare the differences between conditions. MultiExperiment Viewer in the TM4 suite software and MetaboAnalyst were used for visualization and statistical analyses^37,38^. Protein functions, biological processes, and cellular components were identified using Panther^39^. Proteins were differentially analyzed using Venn diagrams^40^. The STITCH database v.5 was used to determine functional protein interaction networks^41^.

### Quantitative RT-PCR

Total RNA from HULEC-5a cells was extracted using TRIzol (Invitrogen) and a PureLink RNA Mini Kit (12,183,020; Invitrogen). A Tetro cDNA Synthesis Kit (BIO-65043; BIOLINE) was used to synthesize cDNA. RT-PCR was performed with specific primers (Table 2) using a SensiFAST STBR^®^ No-ROX Kit (BIO-98005; BIOLINE) and C1000 Touch Thermal Cycler (Bio-Rad). The 2^−ΔΔCt^ method was used to evaluate relative gene expression.

**Table 2:** Human primer sequences for RT-PCR.

Target genes	Forward primer	Reverse primer	Reference
*GAPDH*	5′-CGACCACTTTGTCAAGCTCA-3′	5′-AGGGGTCTACATGGCAACTG-3′	^42^
Proinflammatory chemokine			
*IL8*	5′-CATACTCCAAACCTTTCCACCCC-3′	5′-TCAGCCCTCTTCAAAAACTTCTCCA-3′	^43^
Proinflammatory cytokine			
*IL6*	5′-ATGAGCTCCTTCTCCACAAGCGC-3′	5′-GAAGAGCCCTCAGGCTGGACTG-3′	^43^
Toll-like receptor			
*TLR1*	5′-AGATTTCTTGCCACCCTACTG -3′	5′-GCTCAACCCCAGAAATTTTAG-3′	^44^
*TLR2*	5′-AACTTACTGGGAAATCCTTAC-3′	5′-AAAAATCTCCAGCAGTAAAAT-3′	^44^
*TLR4*	5′-CGAGGAAGAGAAGACACCAGT-3′	5′-CATCATCCTCACTGCTTCTGT-3′	^44^
Permeability markers			
*Caveolin-1*	5′-GAGCTGAGCGAGAAGCAAGT-3′	5′-CAAATGCCGTCAAAACTGTG-3′	^42^
*Claudin-5*	5′-GAGGCGTGCTCTACCTGTTT-3′	5′-GTACTTCACGGGGAAGCTGA-3′	^42^

### Immunofluorescence staining for F-actin

Cells were fixed using a fixative solution (2% paraformaldehyde + 2% glutaraldehyde in PBS) and permeabilized using 0.1% Triton X-100 in PBS, and F-actin and nuclei were stained using phalloidin-iFlour 555 (1:200, ab176156; Abcam, UK) and 4′,6-diamidino-2-phenylindole (DAPI) (D9542; Sigma-Aldrich), respectively.

To detect caveolin-1 and claudin-5, cells were fixed, permeabilized, blocked with 5% BSA for 1 h, and incubated overnight with rabbit anti-caveolin-1 primary antibody (1:500, ab2910; Abcam, UK) or rabbit anti-claudin-5 primary antibody (1:500, ab15106; Abcam, UK). Alexa Fluor 488-conjugated goat antirabbit IgG secondary antibody (ab150085; Abcam, UK) and DAPI were used for immunofluorescence. Cells were visualized using a fluorescence microscope (Nikon DS-Ri2).

### Statistical analysis

Statistical analysis was performed using GraphPad Prism version 7. Data are presented as mean ± SEM. Statistical differences between groups were determined using one-way analysis of variance (ANOVA) with Bonferroni’s post-hoc analysis. Statistical significance was set at *p* < 0.05.

### Supplementary Information

Supplementary Table S1.

## Data Availability

The datasets from the current study are available from the corresponding author on reasonable request.
